# Harnessing Wearable Devices for Emotional Intelligence: Therapeutic Applications in Digital Health

**DOI:** 10.3390/s23198092

**Published:** 2023-09-26

**Authors:** Herag Arabian, Tamer Abdulbaki Alshirbaji, Ramona Schmid, Verena Wagner-Hartl, J. Geoffrey Chase, Knut Moeller

**Affiliations:** 1 Institute of Technical Medicine (ITeM), Furtwangen University, 78054 Villingen-Schwenningen, Germany; 2 Innovation Center Computer Assisted Surgery (ICCAS), University of Leipzig, 04103 Leipzig, Germany; 3 Department of Industrial Technologies, Campus Tuttlingen Furtwangen University, 78532 Tuttlingen, Germany; 4 Department of Mechanical Engineering, University of Canterbury, Christchurch 8041, New Zealand

**Keywords:** digital health, electrocardiogram (ECG), electrodermal activity (EDA), emotion detection, heart rate variability (HRV), machine learning, mental well-being

## Abstract

Emotional intelligence strives to bridge the gap between human and machine interactions. The application of such systems varies and is becoming more prominent as healthcare services seek to provide more efficient care by utilizing smart digital health apps. One application in digital health is the incorporation of emotion recognition systems as a tool for therapeutic interventions. To this end, a system is designed to collect and analyze physiological signal data, such as electrodermal activity (EDA) and electrocardiogram (ECG), from smart wearable devices. The data are collected from different subjects of varying ages taking part in a study on emotion induction methods. The obtained signals are processed to identify stimulus trigger instances and classify the different reaction stages, as well as arousal strength, using signal processing and machine learning techniques. The reaction stages are identified using a support vector machine algorithm, while the arousal strength is classified using the ResNet50 network architecture. The findings indicate that the EDA signal effectively identifies the emotional trigger, registering a root mean squared error (RMSE) of 0.9871. The features collected from the ECG signal show efficient emotion detection with 94.19% accuracy. However, arousal strength classification is only able to reach 60.37% accuracy on the given dataset. The proposed system effectively detects emotional reactions and can categorize their arousal strength in response to specific stimuli. Such a system could be integrated into therapeutic settings to monitor patients’ emotional responses during therapy sessions. This real-time feedback can guide therapists in adjusting their strategies or interventions.

## 1. Introduction

The use of artificial intelligence (AI) in daily activities has become mainstream in recent years. Advances in technology have paved the way for computationally powerful machine learning models to cement the foundations for the future of the industrial and healthcare domains. The adoption of AI in the health sector holds a lot of potential, from patient diagnostics to health monitoring and, in some cases, treatment itself [1].

Emotional intelligence strives to bridge the gap between human and machine interactions. The application of such systems varies and is becoming more prominent as healthcare services work to provide more efficient care through the utilization of smart digital health apps. One application in digital health is for the incorporation of emotion recognition systems as a tool for therapeutic interventions. Emotion classification is currently being developed as a component in a closed-loop system [2] designed to aid in the therapeutic intervention of people with autism spectrum disorder (ASD).

ASD is a neuro-developmental condition that affects a person’s social skills by impairing their interaction, communication, behaviors, and interests [1,3,4]. The condition often results in more health problems due to isolation and unemployment (or reduced employment), which can lead to depression and anxiety [4]. Estimates reveal that 1 out of 59 people are affected by ASD, thus comprising ~1~2% of the general population [4,5].

Emotions can be identified by three main components: 1—facial expressions; 2—speech and voice patterns; and 3—physiological signals. Emotion recognition perception is distributed as 55% facial, 35% speech, and 10% physiological signals [6]. Although facial expressions and speech patterns hold the majority for emotion determination, limited access to these data in real time in daily life makes them less convenient than physiological signals. Physiological signals can be accessed through electronic wearable devices (EWD), such as smart watches, which are increasingly prevalent and are directly associated with health management [7]. Equally, screen time, including smart phone, TV, and computer usage, stands at 28.5 ± 11.6 h a week [8]. Even if a small portion of screen time is allocated to using a health app, the data collected would still be fewer than the level of data from EWDs. Physiological signals often used to measure emotional and cognitive reactions include electrodermal activity (EDA) and electrocardiogram (ECG) [9,10,11]. Hence, physiological signals were selected for emotion detection in this study.

For electrodermal activity, the parameters of the frequency of non-specific skin conductance responses (NS.SCR) and the skin conductance level (SCL) are frequently used. This is one of the most common measures used in psychophysiology and includes a wide range of applications, such as emotional reactions, attention examination, and the processing of information. EDA is measured by applying a small current through a pair of electrodes that are placed on the surface of the skin [12]. Two mechanisms contribute to the EDA measurement: 1—sweat secretion and 2—selective membrane activity in the epidermis. The more sweat produced, the more conductive the path becomes; as a result, the resistance decreases and therefore a change is observed in the EDA.

ECG is one of the most widely used non-invasive clinical diagnostic tools, providing a clear observation of the heart’s electrical behavior [13]. ECG records the electrical activity transmitted through the body by means of electrodes attached to the skin. Another relatively simple derivation option is the use of a chest belt. This electrical activity is the result of the heart’s depolarization to induce contraction at each beat [14]. The measurements are analyzed through the QRS wave complex, and subsequently the heart rate (HR) is derived from peak to peak, e.g., RR interval, of the ECG recording across a specific time frame. The use of ECG monitoring has increased in recent years, thanks in part to the advancement of wearable devices, such as smart watch technology or fitness trackers, and people’s often high adherence to their use for the monitoring of daily activity and workout routines in a lifestyle focused on well-being and healthy aging.

The data used in this article were collected from a separate collaborative study conducted on emotion induction methods’ influence on recognition [15]. The ground truth, defined as the subjectively perceived valence and arousal of each emotional category, was assessed using the self-assessment manikin (SAM) [15,16]. The data were gathered from EDA and ECG sensors attached to the non-dominant hand (thenar and hypothenar) and chest, respectively.

In this study, the EDA—more specifically, the SCL—and ECG signals, i.e., HR and heart rate variability (HRV) were analyzed for emotional stimulus trigger marks and assessed for the different emotional reaction stages and intensity of arousal using signal processing and machine learning techniques. Features of interest, required for the machine learning algorithm, were extracted from the data by applying different signal processing methods. To evaluate the outcome of the predictions, different evaluation criteria were used. The aim of this study was to disclose the effectiveness of physiological signals—in this case, EDA and ECG—in characterizing emotional stimuli reactions and identifying their stages and arousal strength.

The paper is organized with the following structure. Section 2 describes the methods used, data description, signal processes, network architecture, and analysis criteria. Key results are highlighted in Section 3, with their respective discussions rendered in Section 4. The conducted ablation studies are mentioned in Section 5, and a conclusion is drawn in Section 6.

### Related Work

The challenges of detecting and recognizing human emotions have yielded different approaches and techniques, with a recent trend towards machine learning strategies to solve the problem. A recent search for “emotion recognition facial” and “emotion recognition physiological signal” on PubMed revealed the concentration of research works towards facial recognition (4825 articles), rather than physiological signals (191 articles), for emotion recognition, with a ratio of ~25:1 over the last 5 years [17].

In Kakuba S. et al. (2022) [18], an attention-based multi-learning model (ABMD) utilizing residual dilated causal convolution (RDCC) blocks and dilated convolution (DC) with multi-head attention is proposed for emotion recognition from speech patterns, achieving 95.83% on the EMODB dataset, with notable robustness in distinguishing the emotion of happiness. In Yan Y. et al. (2022) [19], an AA-CBGRU network model is proposed for speech emotion recognition that combines spectrogram derivatives, convolutional neural networks with residual blocks, and BGRU with attention layers, showing improved weighted and unweighted accuracy on the IEMOCAP sentiment corpus. In Khaireddin Y. et al. (2021) [20], a popular VGG network architecture was deployed with fine hyperparameter tuning to achieve state of the art results on the FER2013 [21] dataset. A shallow dual network architecture was introduced in Mehendale N. (2020) [22], with one framework removing background noise while the second generated point landmark features, achieving recognition accuracies of up to 96% on a combined dataset. Zhao X. et al. (2017) [23] proposed a novel peak-piloted GoogleNet [24] network architecture in which the peak and non-peak emotional reaction was considered from an image sequence, with tests on the OULU-CASIA [13] database achieving up to 84.59% accuracy.

In Kim Y. et al. (2021) [25], a facial image threshing (FIT) machine for autonomous vehicles’ facial emotion recognition (FER) is introduced, utilizing advanced features from pre-trained facial recognition and the Xception algorithm, resulting in a 16.95% increase in validation accuracy and a 5% improvement in real-time testing with the FER 2013 dataset compared to conventional methods. In Canal F. et al. (2022) [26], a survey was conducted that reviewed 94 methods from 51 papers on emotion expression recognition from facial images, categorizing them into classical approaches and neural networks, finding slightly better precision for the classical methods but with lesser generalization; this work also evaluated the strengths and weaknesses of popular datasets. In Karnati M. et al. (2023) [27], a thorough survey of deep learning-based methods for facial expression recognition (FER) is provided, which discusses their components, performance, advantages, and limitations, while also examining relevant FER databases and pondering the field’s future challenges and opportunities.

Although the facial features provide a more distinguishable analysis of the emotional response of a person, the acquisition of the data is somewhat cumbersome. The relevant and appropriate feature extraction from facial expressions in images is also disputed. In particular, it is often not robust to differences in complexion, culture, and ethnicity.

Physiological signals provide more continuous real-time monitoring compared to facial expressions. In comparable studies [28,29,30,31,32,33,34,35], the impact of using physiological signals for emotion detection and subsequent recognition is highlighted. Shukla J. et al. (2021) [28] assessed and evaluated different techniques for EDA signals and determined the optimal number of features required to yield high accuracy and real-time emotion recognition. A fine hyperparameter-tuned convolutional neural network was developed in Al Machot F. et al. (2019) [29] for use in assisted living environments using EDA signals to recognize emotions. The designed model improved the robustness of two established datasets, achieving accuracies of 78% and 82% on the MAHNOB [36] and DEAP [37] datasets, respectively, for subject-independent recognition. In Veeranki Y. R. et al. (2021) [30], different time–frequency signal analysis methods are implemented on the EDA signal and combined with machine learning techniques for emotion recognition, reaching area under the curve (AUC) accuracies of 71.30% on the DEAP [37] database. In Wenqian L. et al. (2023) [38], a review was conducted on emotion recognition and judgment using physiological signals like EEGs, EDA, ECGs, and EMG, discussing their technological applications and the effects achieved and providing a comparative analysis of different signal applications, along with considerations for future research.

Heart rate (HR) monitoring, using smart watches, is often applied when following up on pre-existing health conditions or tracking workout routines for athletes [7]. However, other applications, such as stress level detection and emotion recognition, are also studied [31,39]. In Shu L. et al. (2020) [31], HR signals recorded by a smart wearable device were assessed for the recognition of paired emotions using machine learning models. The approach achieved accuracy of 84% for three emotional states’ classification, using a gradient boosted decision tree algorithm on the collected dataset. Zhang Z. et al. (2016) [35] took a different approach to recognizing emotions, using the accelerometer data from wearable devices. The results revealed accuracy of 81.2% in classifying three emotional categories, using a support vector machine (SVM) with a radial basis (RBF) kernel function as a classifier.

A combination, more commonly known as fusion, of more than one signal for emotion recognition has also been studied, with promising results. Greco A. et al. (2019) explored the fusion of both EDA signals and speech patterns to improve arousal level recognition, yielding a marginal classifier improvement of 11.64% using an SVM classifier with recursive feature elimination [32]. Du G. et al. (2020) investigated the combination of facial expressions and HR for emotion recognition in gaming environments, increasing the recognition accuracy by 8.30% [33]. In Fernández-Aguilar L. et al. (2019) [34], the fusion of EDA signals and HR variability (HRV) was used for emotion classification, achieving 82.37% overall accuracy for both young and elderly age groups combined, for seven emotion classes, using an SVM classifier with a quadratic kernel.

Hence, both EDA and ECG signals were used in the present study for emotion identification and its subsequent arousal level determination. This study was distinct from prior research as it did not focus on identifying the relative emotional response but rather the ability to identify the physiological reaction and its subsequent arousal intensity. This approach offers a more detailed understanding of an individual’s level of engagement with the presented stimuli.

## 2. Materials and Methods

### 2.1. Database Description

The data used in this research were collected as part of a study on emotion induction techniques, under controlled laboratory conditions [15]. Physiological measurements of ECG and EDA were recorded, along with videos of the facial expressions. In total, 24 subjects (10 male, 14 female), from different age groups, volunteered.

The experiment consisted of having the subjects sit and watch a slideshow recording containing 7 different image stimuli, comprising the six basic emotions of anger, disgust, fear, happiness, sadness, and surprise, and a seventh neutral category. Each stimulus was applied for 30 s, designed to induce an emotional reaction, followed by a rest time of 1 min between each stimulus. After the rest period, subjects were asked to reflect for a period of 30 s on a situation in their lives where such an emotional trigger had occurred (autobiographical recall), followed another rest period of 1 min. Subjects also assessed each stimulus using the SAM [16], where this information was used as ground truth for system development. A more detailed description of the experiment can be found in Schmid et al. [15].

**Figure 1:**
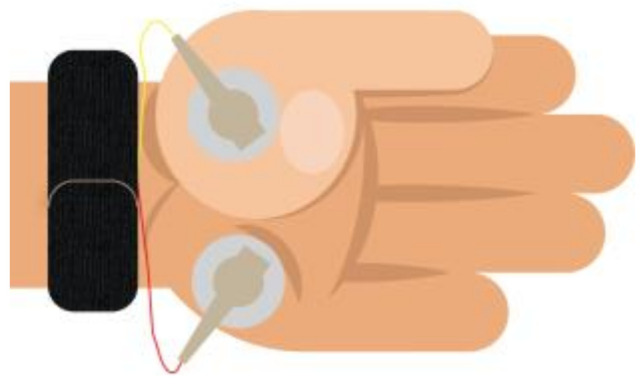
Graphic illustration of the EDA sensor strap and electrode placement on the non-dominant hand of the subject.

Physiological signals were recorded from two sensors on the hand and chest. For the ECG, the “EcgMove4” sensor (Movisens GmbH, Karlsruhe, Germany) with a dry electrode chest belt was used. The “Ecg-Move4” records ECG signals at a rate of 1024 Hz and 12-bit resolution with an input range of 560 mV [40]. To measure EDA, the “EdaMove4” sensor (Movisens GmbH, Karlsruhe, Germany) was used. The “EdaMove4” sensor was attached to the subject’s non-dominant wrist with the two electrodes placed on the palm (thenar and hypothenar), as depicted in Figure 1. The EDA sensor records at a sample rate of 32 Hz with a 14-bit resolution and an input range of 2 to 100 μS [41].

The collected dataset consisted of 24 ECG and EDA signals. For system development, the signal sequences were annotated for each subject and signal, based on the used emotional categories (anger, disgust, fear, happiness, neutral, sadness, and surprise) and the participants’ assessment using the SAM [16]. The following measurement times (recording sequences) were used for each emotional category: (a) during image presentation (30 s), (b) rest period after image presentation (60 s), (c) during autobiographical recall (30 s), (d) rest period after autobiographical recall (60 s), and (e) a baseline measurement recorded at the beginning of the experiment. The arousal level was retrieved from the SAM assessments using a 9-point scale (from 1—low arousal to 9—high arousal) based on pictograms.

In this study, a two-class classification model was first established to classify the state of the signal as either an emotion or resting stage. Afterwards, a three-class classification model was developed to identify the arousal strength of the detected emotion. The 9-point arousal scale was converted to a three-class arousal strength by setting the values 1 to 3 as low, 4 to 6 as mid, and 7 to 9 as high. Table 1 represents the arousal scale conversion. The baseline and emotion classes consisted of recordings of 30 s, while the rest period had a 60 s duration.

### 2.2. System Methodology

**Figure 2:**
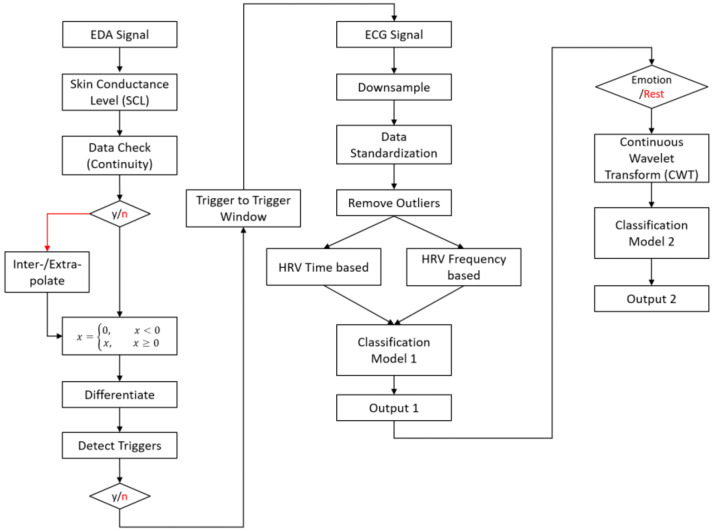
Flow chart of the system workflow for EDA and ECG signal analysis. The EDA analysis path is used to detect the changes in signal activity. The trigger period is then used for the ECG signal path analysis and classification of the emotional state and arousal strength. The red font indicates a flow process that was rejected and removed from further processing, unless illustrated otherwise.

The workflow of the proposed system in real-time applications is depicted in Figure 2. The physiological signal analysis was separated into two paths, one for EDA and another for ECG. The EDA data obtained from the experiments had to be pre-processed to address disturbances, such as invalid measurements and signal discontinuity, during data gathering and post-processing, which included skin conductance level (SCL) calculation. Signals were then processed to determine emotional stimulus trigger time stamps. This key information was used in conjunction with the ECG signal classification model.

The ECG signals collected were then separated into signal snippets based on the information from the EDA analysis. The ECG signal was first down-sampled and then standardized for a consistent stimulus activity period between the subjects. This processing was performed to address data synchronization issues. Outliers were then removed and heart rate variability (HRV) calculated using two different time- and frequency-based methods [42]. The HRV was then used as input to classification model 1, designed to find a pattern within the data and classify the two states of the subject, emotion and rest. Next, the emotion signal was passed through a continuous wavelet transform (CWT) to convert the signal into an image, and then passed through classification model 2, where the emotion signal arousal strength was classified.

### 2.3. Signal Processing

#### 2.3.1. EDA Signal Processing

**Figure 3:**
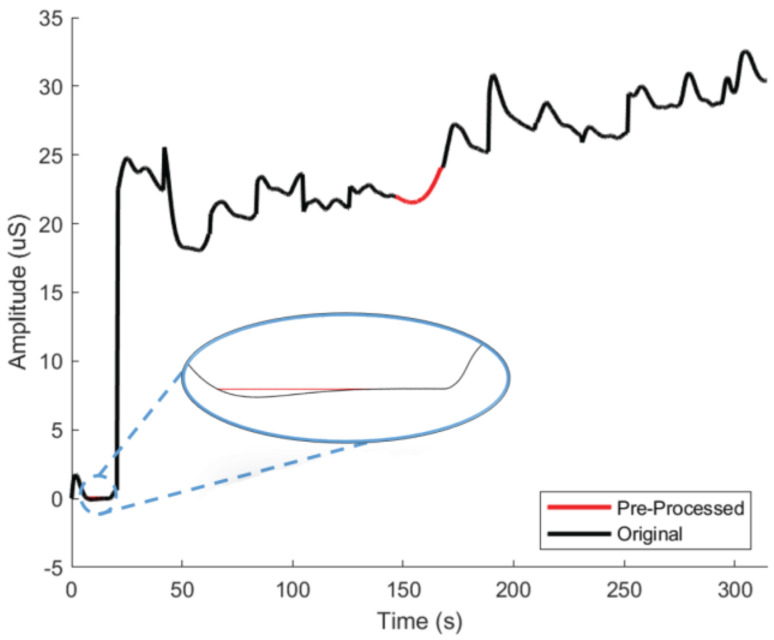
Sample of SCL signal before and after the pre-processing stage. The black line shows the original signal, and the red lines represent the signal output after pre-processing.

Given the placement positions of the electrodes and sensor for EDA data collection, inconsistencies and noise were unavoidable. To counter these disturbances, the SCL output derived from the EDA signal underwent a pre-processing stage. During the pre-processing stage, the SCL signal was scanned for missing data, such as not-a-number (nan) errors, for each subject. If a discontinuity was detected, piecewise cubic spline interpolation was used to fill the gap. After this, a threshold was set to change any non-physiological value below zero to zero to counteract false measurements. Figure 3 shows an example before and after pre-processing.

To detect emotional stimulus trigger marks from the SCL data, a second-order derivative was performed to determine the deflection points in the signal. The output was then used to extract the peaks, which represent the instance where a change in the EDA is observed. The time frame between two consecutive trigger marks was later used as the basis for the ECG signal snippet.

#### 2.3.2. ECG Signal Processing

**Figure 4:**
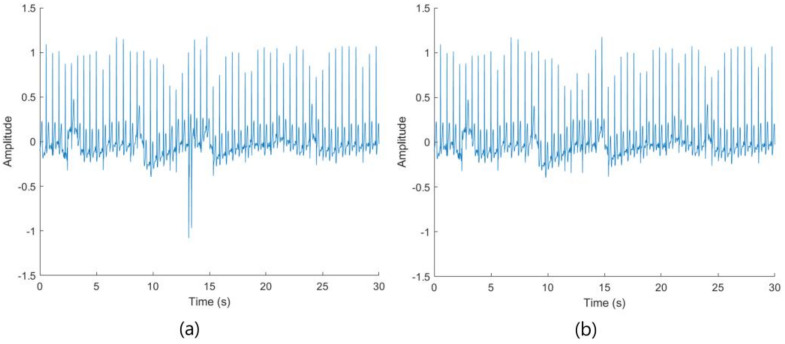
ECG signal of baseline measurement of a subject. (**a**) Signal before (**left**) and (**b**) after (**right**) outlier removal.

The ECG signal was first down-sampled from 1024 to 256 Hz, and then subdivided into 29 shorter signals representing the stimulus reactions from the experiment, the 14 emotions (7 from visual stimulus and 7 from autobiographical recall), the 14 corresponding rest stages, and a baseline measurement at the beginning of the experiment. Next, outliers detected in the signals were removed by applying a 1 s sliding window with a stride of one second to extract the minimum (min) and maximum (max) values across each stimulus response. For each subject, the mean of the min and max was calculated in the respective window frame and a threshold value set, so that any min and max value less than and greater than, respectively, 2.5 times the mean min and max value was tagged for removal. The tagged signal was then replaced with either its predecessor or successor of the same length depending on the position of the highlighted signal. The algorithm used for outlier removal is described in Appendix A. An example of the outlier removal algorithm applied to the baseline measurement is shown in Figure 4.

After removing the outliers from the raw ECG signal, the RR intervals were calculated between the peaks of the QRS complex wave. When analyzing the output of the RR intervals, different outliers were observed. Therefore, a separate outlier removal algorithm was implemented on the RR intervals using a generalized extreme Studentized deviate test [43] and a modified Akima cubic Hermite interpolation [44,45] to fill gaps caused by the discarded information.

Outliers were removed to enhance the accuracy and robustness of the analysis. Outliers can distort underlying trends in the data, leading to potentially misleading results. By excluding these anomalies, the analysis benefits from a more consistent and representative dataset, thereby ensuring the validity of the conclusions drawn.

### 2.4. Feature Extraction

To achieve robust prediction, meaningful features need to be extracted. Since the ECG information was used to classify the different stages of the response, the heart rate variability (HRV) was selected as a relevant feature. The HRV can be calculated using time- or frequency-based techniques. In total, eight features were selected as input to the classifier, 4 time-based and 4 frequency-based. Time-based HRV features extracted comprised 1—the root mean square of successive differences between heartbeats (RMSSD), 2—the standard deviation of the RR intervals measured in ms (SDNN), 3—the mean of the RR intervals (RR_Avg), and 4—the heart rate (HR). Frequency-based HRV measures comprised 1—the high-frequency power (HF), 2—the low-frequency power (LF), 3—very low-frequency power (VLF), and 4—the ratio of high-frequency to low-frequency power (HF2LF).

These features were selected since HRV captures the variability between successive heartbeats and offers insights into the autonomic nervous system (ANS), which is integral to emotional processing. Time-based HRV features measure overall heart rate variability and its rapid changes, with alterations indicating different emotional responses. In the frequency-based HRV, the balance between low-frequency and high-frequency components can reflect shifts in emotional states, with specific patterns potentially distinguishing emotions like joy from sadness or anger. Overall, HRV serves as a valuable tool in deciphering the body’s autonomic responses to emotions, aiding in understanding emotional regulation and processing.

#### 2.4.1. Time-Based HRV

The RMSSD is calculated as the difference in time between two consecutive R waves in milliseconds (ms) over a set period of time. In this study, 30 and 60 s time windows were chosen for the RMSSD for emotion and rest, respectively, as these perform as well as the 5 min period [42,46]. The computation of the RMSSD, where *RR* represents the time interval between R peaks and *N* is the total number of RR intervals, is defined as

(1) $$\mathrm{RMSSD}=\sqrt{\frac{1}{N-1}\overset{N-1}{\underset{i=1}{\sum }}{\left(RR_{i+1}-RR_{i}\right)}^{2}}$$

The SDNN is the standard deviation of the *RR* time intervals over the length of the signal and is defined as

(2) $$\mathrm{SDNN}=\sqrt{\frac{1}{N}\overset{N}{\underset{i=1}{\sum }}{\left(RR_{i}-\mu \right)}^{2}}$$

where μ represents the mean of the *RR* intervals in ms.

The RR_Avg feature is calculated as the mean of the *RR* intervals, and *HR* is calculated as the number of RR intervals in a 60 s time window:

(3) $$HR=\frac{60*1000}{\mu }$$

#### 2.4.2. Frequency-Based HRV

The frequency domain can be used to separate HRV into power in different frequency ranges [42]. In this study, the Lomb–Scargle power spectral density [47] was used to estimate the periodogram and frequencies of the given signal. Afterwards, the output was separated into the three frequency ranges of HF, LF, and VLF. The HF2LF is calculated as the ratio of HF to LF. The following frequency limits [42] were used for the calculation:

- HF: 0.15–0.4 Hz;
- LF: 0.04–0.15 Hz;
- VLF: 0.003–0.04 Hz.

The sum square energy was calculated for each of the HF, LF, and VLF, as follows:

(4) $$x\_Feat=\sum_{i}P\left(f_{i}\right)\;\;\;\;\forall \left(n\;<f_{i}>\;m\right)$$

where P represents the periodogram data, f the frequency, n the lower limit, and m the upper limit of the corresponding frequency range.

#### 2.4.3. Continuous Wavelet Transform (CWT)

**Figure 5:**
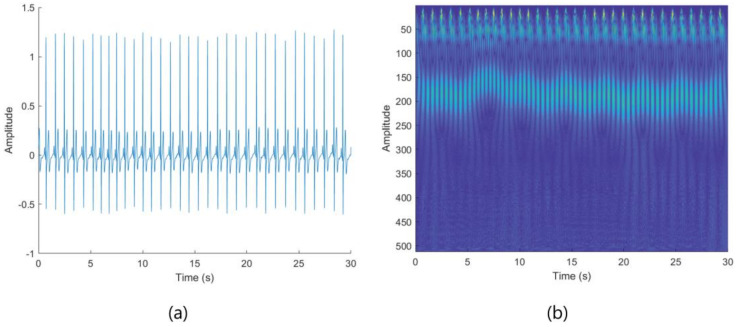
ECG signal of emotion measurement of a subject. (**a**) Original signal (**left**) and (**b**) signal after applying CWT (**right**).

The CWT was used to extract features for the classification of the emotions’ arousal strength. A sampling frequency of 256 Hz was used with a scale range of 1 to 512, a time bandwidth of 0.234, and a Morlet wavelet [48]. Figure 5 shows the output (Figure 5b) from the CWT with a given ECG signal snippet input (Figure 5a).

### 2.5. Classification Models

#### 2.5.1. Emotion Detector

To distinguish a signal’s emotion state, divided into either emotion or rest, from the gathered features, a machine learning algorithm was adopted. Different models were tested and the results are presented in the ablation study in Section 5.1, and the best-performing one was selected. The support vector machine (SVM) classification model was thus used to classify this two-class system. The SVM classifier has many strong points suitable for this task, as they are versatile, robust to overfitting, and effective in high-dimensional spaces [49,50].

The hyperparameters of the SVM were optimized using a Bayesian optimization function for 100 iterations with a 5-fold cross-validation scheme. The optimized and selected hyperparameters are described in Table 2. The model classified the signal as either emotion or rest based on the predicted probability. The input features were normalized to the range of 0 and 1 across each observation.

#### 2.5.2. Arousal Strength Classifier

After identifying a signal as an emotion, it was passed through a CWT to convert the signal into an image before entering classification model 2, to determine the arousal strength of the given emotional response. To classify the image into one of the three arousal strength classes, deep learning convolutional neural network (CNN) models were utilized. Different CNN architectures were tested, the results of which are given in the ablation study in Section 5.2. The best-performing model was selected for the classification.

The ResNet-50 [51] architecture with initial pre-trained weights, trained on the ImageNet dataset, was used for model training. The last fully connected layer of the architecture was replaced such that the output was set to 3, which represents the number of classes for classification. Weighted cross-entropy was used for the loss function:

(5) $$loss=-\frac{1}{N}\overset{N}{\underset{n=1}{\sum }}\overset{K}{\underset{i=1}{\sum }}w_{i}T_{ni}\ln Y_{ni}$$

$$w_{i}=\frac{N}{m_{i}}$$

where N is the total number of observations, K is the total number of classes, and $w_{i}$ is the weight at class i. $m_{i}$ is the number of observations for class i, and T is the GT value for the predicted value T. Table 3 shows the different training options used for model training.

### 2.6. Evaluation Criteria

To evaluate the performance of the different systems, different metrics were selected. To assess the trigger mark detection from the SCL signal, the root mean squared error (*RMSE*) was used:

(6) $$RMSE=\sqrt{\frac{1}{N}\overset{N}{\underset{i=1}{\sum }}{\left(x_{i}-{\hat{x}}_{i}\right)}^{2}}$$

where *N* represents the total number of trigger marks, *x* the annotated trigger, and $\hat{x}$ the predicted trigger at a certain time.

The emotion detector and arousal strength classifier models were evaluated using a 5-fold Monte Carlo cross-validation scheme. Performance was based on the mean of the accuracy and F1-score over the 5 folds. The Fβ-score is calculated as follows:

(7) $$F\beta \_\mathrm{score}=\frac{\left(\left(1+\beta^{2}\right)*\mathrm{Precision}*\mathrm{Recall}\right)}{\left(\beta^{2}*\mathrm{Precision}\right)+\mathrm{Recall}}$$

(8) $$\mathrm{Precision}=\frac{\mathrm{TP}}{\mathrm{TP}+\mathrm{FP}}$$

(9) $$\mathrm{Recall}=\frac{\mathrm{TP}}{\mathrm{TP}+\mathrm{FN}}$$

where the β. is a coefficient used to weight the precision, and, in this work, β is set to 1 to have a weighted balance between precision and recall. In Equations (8) and (9), TP stands for the true positive, FP for false positive, and FN for false negative predictions. For the second classification model (arousal strength identification), the TP accuracy was used to assess the model performance.

## 3. Results

### 3.1. Dataset Distribution

Table 4 represents the original and selected datasets’ class distribution. The different emotional classes of anger, disgust, fear, happiness, neutral, sadness, and surprise were combined to form one class under the representation of emotion. Therefore, the two-class system consisted of 266 observations for emotion and 266 observations for rest from the selected dataset.

Table 5 displays the distribution of the arousal levels from the SAM assessments. As described in Section 2.1, a three-class system was established from the nine-point SAM and the distribution of the dataset was 84 for low, 121 for mid, and 61 for high arousal strength. The arousal strength labels were then randomly split into a training and testing set with a ratio of 90% training, with 240 observations, and 10% testing, with 26 observations, such that at least one observation from each nine-point SAM class was present in the testing set.

### 3.2. SCL Trigger Point Detection

**Figure 6:**
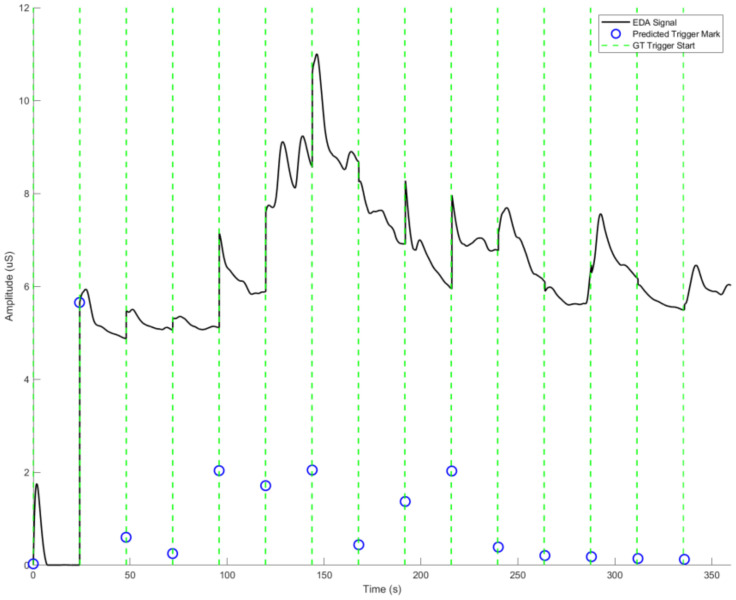
Sample of an SCL signal for image stimulus with predicted and ground truth (GT) trigger marks of a subject from the dataset. The blue circles represent the predicted trigger points, green dashed lines the GT, and the black solid line the SCL.

The first phase of the system workflow demonstrated the efficient detection of the trigger marks form the SCL signal, as observed in Figure 6. The strategy and steps adopted were able to achieve an RMSE value of 0.9871 for all the trigger mark time stamps, for each stage of emotion and rest, at both emotion induction methods, for all subjects.

### 3.3. ECG Signal Identification

#### 3.3.1. Emotion and Rest Detection

**Figure 7:**
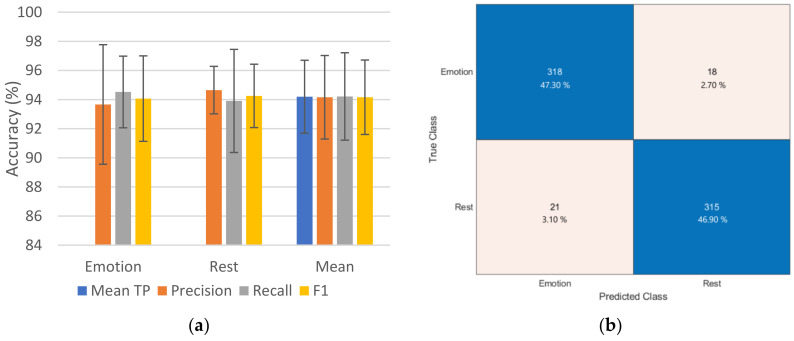
Evaluation results of the performed 5-fold cross-validation on the classification model for emotion and rest detection. (**a**) Mean precision, recall, and F1-score for each class over all 5 folds and their means, along with mean TP accuracy over both classes and folds, with the standard deviations depicted as error bars. (**b**) Confusion chart summed across all the 5-fold validation sets; blue regions indicate the true positive (TP) of the corresponding class.

In Figure 7a, the average TP accuracy across both classes, as well as the average precision, recall, and F1-score accumulated over the five folds, are displayed. Figure 7b also shows the aggregated confusion matrix over all five folds for both the emotion and rest classes. The model achieved mean TP accuracy of 94.19% ± 2.50, with mean precision of 94.16% ± 2.87, a recall mean of 94.21% ± 3.00, and a mean of 94.16% ± 2.55 for the F1-score over all five folds and classes. The confusion chart revealed that the model had a misclassification rate of 5.36% and 6.25% for the emotion and rest classes, respectively.

#### 3.3.2. Arousal Detection

**Figure 8:**
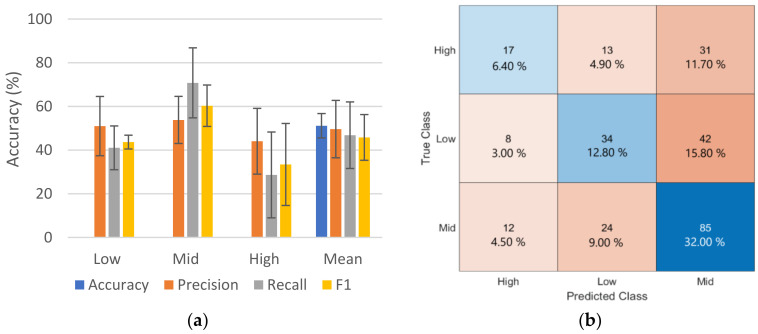
Evaluation results of the 5-fold cross-validation from the classification model for arousal strength classification. (**a**) Mean of the precision, recall, and F1-score over the 5 folds and the mean over all the classes along with the TP accuracy. The standard deviation is depicted as error bars. (**b**) Confusion chart summed across all the 5-fold validation sets; blue regions indicate the true positive (TP) of the corresponding class.

The results from the classification of the emotions’ arousal strength are represented in Figure 8. The mean of the precision, recall, and F1-score over all five folds for each class is displayed in Figure 8a, along with the mean and mean TP accuracy, whereas, in Figure 8b, the summed confusion matrix over the five folds is depicted. The proposed model showed some fluctuations in performance, reaching mean TP accuracy of 51.14% ± 5.58 over the five folds. The mid arousal strength class showed the best performance among the classes, achieving an F1-score of 60.31% ± 9.48, while the high arousal strength class performed the poorest, with an F1-score of 33.41% ± 18.77. The best-performing model out of the five trained models achieved mean TP accuracy of 60.37% over all the classes.

The confusion chart shows that the majority of the misclassifications of the low and mid arousal strengths were linked to the mid arousal strength class with a rate of 50.81% and 50% for the high and low classes, respectively.

## 4. Discussion

As observed in Table 4, the selected dataset was smaller than the original, with a reduction of 20.83%. This reduction resulted from a first-stage signal analysis on the original ECG signal, where data from five subjects revealed inconsistencies in the recording. As a consequence, these samples were removed from further processing.

The distribution in Table 4 also demonstrates there was no bias towards a particular class in the two-class system. Thus, there was equal representation during the training process. However, in Table 5, a bias in the data towards the class of mid arousal strength is revealed, having a rate of 45.49% from the total distribution, with 31.58% for low and 22.93% for high. This data imbalance was countered with a class-weighted loss function, as described in Section 2.5.2. This ensured the fair representation of each of the arousal strength classes during model training.

The efficacy of the proposed model in distinguishing between the two classes of emotion and rest is highlighted in Figure 7. The results indicate that the selected features, and HRV specifically, have suitable embedded information for the task of distinguishing between an emotion or calm or resting state. The robustness of the model at this stage makes further processes throughout the workflow pipeline more efficient. Thus, overall errors will be more sensitive to the model’s capability in identifying the strength of a detected emotion’s arousal.

The results in Figure 8 reveal the difficulty in identifying the different arousal strengths from the given dataset. One contributing factor to the heightened performance of the mid arousal strength could be the inherent human uncertainty or variability surrounding the projection of mid-range arousals. Contrary to real-life scenarios, where extreme emotions tend to offer clearer cues, the model appears particularly adept at navigating the nuances of these intermediate arousal strengths, possibly because of the complexities and ambiguities that humans exhibit when expressing them.

In addition, the use of deep learning models is a high-dimensional problem and requires significantly large datasets. Another contributing factor to this low performance was linked to the data imbalance, as well as the limited number of total observations. The data augmentation technique of signal oversampling was not adopted as it would have led to the model overfitting on the data.

The low representation of the high arousal strength class also indicates that the subjects were not strongly impacted by the experiment’s stimuli. Thus, no significant change in their ECG signal was present. Indeed, when examining the recorded videos, which were synchronized with the physiological signal measurements, minimal to no change in the person’s facial expressions was observed. It is thus worth noting the need for potentially more extensive tests to ensure that this state is better represented in the data, if possible.

Further, the dataset used in this study was composed of real human reactions to stimuli perceived to trigger the corresponding emotional response. As a result, the complexity of classification increased, since each person behaved differently towards the same stimuli. Equally, the physiological signals also differed from one person to the other depending on a wide range of factors, which in turn influenced the acquired features.

In the broader context of emotion recognition, this research underscores the potential of physiological signals, specifically electrodermal activity (EDA) and electrocardiogram (ECG) data, in accurately detecting emotions and assessing arousal strength. The notable emotion detection accuracy of 94.19% achieved by emphasizing key descriptors from heart rate variability (HRV) signifies a substantial advancement in the utilization of these physiological markers. The proposed pipeline, with its real-time application capability, highlights the emerging role of wearable devices in advancing the realm of digital health therapeutics. Additionally, by incorporating a system that can be integrated into therapeutic settings, the research paves the way for more personalized and adaptive therapeutic interventions. The methodology, especially when compared to previous works, showcases the efficacy of combining multiple physiological markers. Thus, this study adds a pivotal dimension to the ongoing discourse in emotion recognition by emphasizing real-time, wearable-device-driven insights, bridging the gap between laboratory findings and real-world therapeutic applications.

As with any research, certain limitations of the study should be noted. Limitations include no optimization on the signal window length for HRV feature extraction, no hyperparameter tuning on the CWT, and no model explicability analysis. It should be noted that the signal window length for HRV feature extraction was not optimized, which could have influenced the accuracy of the HRV features derived. Additionally, the absence of hyperparameter tuning for the continuous wavelet transform (CWT) suggests that the decomposition of the signal into its constituent frequencies might not have been at its optimal state, potentially impacting the precision of the feature extraction. Furthermore, without a detailed explicability analysis, the underlying rationale behind the model’s decisions remained challenging to decipher, which might limit its practical application. These factors collectively may constrain the generalizability of the findings.

The focus of future work will be to tackle some of these limitations by performing an ablation study on the window length. An optimization function will be implemented to tune the CWT hyperparameters. To evaluate the explicability of the model, different techniques will be employed and an evaluation metric established for a quantitative measurement.

## 5. Ablation Study

### 5.1. Traditional Classifier Algorithm Selection

To assess the performance and impact of the classification model on the given dataset for emotion and rest classification, different traditional machine learning classifiers were tested. The tested models were trained using the same features and their hyperparameters optimized using the same strategy described in the Methods section, with a 5-fold cross-validation scheme.

Table 6 represents the mean results over the 5 folds on each of the tested models over all the classes. As highlighted, the SVM model with optimized parameters performed the best overall. This indicates that it was able to create a more robust separable feature space than the other tested models.

### 5.2. Network Architecture Influence

A convolutional neural network architecture has a strong effect on the outcome of the model training process. In this study, five different architectures of Alexnet [52], VGG16 [53], GoogleNet [24], EfficientNetb0 [54], and SqueezeNet [55], with initial pre-trained weights, trained on the ImageNet dataset, were trained and analyzed for arousal strength classification using the same training options defined in Section 2.

Each architecture has uniqueness and brings a key strength to the model training process. VGG16 demonstrated that stacking small filters can be as effective as having larger receptive fields with fewer parameters. GoogleNet allows for efficient multi-scale processing by using filters of different sizes in parallel, capturing patterns at various scales. EfficientNetb0 scales all three dimensions of depth, width, and resolution together, in a balanced manner, resulting in efficient high-performing models. ResNet50 allows the network to skip certain layers and reduces the problem of gradient vanishing. SqueezeNet is lightweight and suitable for edge devices with limited computational power and is designed to reduce the number of parameters without a significant loss in accuracy. AlexNet allows the use of grouped convolutions to reduce the computational demand and promote diverse feature extraction.

Table 7 showcases the mean TP accuracy results over all 5 folds and classes for each model architecture. As can be seen, the ResNet50 architecture achieved the best performance, highlighting its ability to learn relevant descriptive features for arousal strength classification.

## 6. Conclusions

This research used physiological signals for emotion detection and arousal strength identification and a pipeline for real-time applications is proposed. The proposed workflow emphasizes the contributions of wearable devices in advancing digital health therapeutics. Such a system could be integrated into therapeutic settings to monitor patients’ emotional responses during therapy sessions. This real-time feedback might be developed into a guide for therapists in adjusting their strategies or interventions. Changes in electrodermal activity (EDA) are first identified and this information is used to reinforce data gathered from the electrocardiogram (ECG) to determine the state of the individual, differentiating between a neutral, calm or rest, or emotional state. Subsequently, the arousal strength of any detected emotional state is classified. The proposed model pipeline was able to achieve emotion detection accuracy of 94.19% with statistical relevance by focusing on key descriptors from the heart rate variability (HRV) features extracted from the ECG signal. Classification accuracy of 51.14% was achieved for the arousal strength identification, which was impacted by significant variability through the mid-range arousal states. Given the complexity of identifying real reactions to emotional stimuli, coupled with the limited amount of data, the proposed approach achieved compelling results, particularly in comparison to prior works and research using more measured input signals. Further analysis and enhancements to the models are planned for future work, including the acquisition of a new dataset along with real-time tests.

## Figures and Tables

**Table 1:** Arousal scale conversion.

Scale	Arousal								
9-Point	1	2	3	4	5	6	7	8	9
3-Class	Low			Mid			High		

**Table 2:** Selected hyperparameters for classification model 1 following the results of the optimization process.

Hyperparameter	
Multi-class coding	One vs. One
Data standardization	False
Kernel function	Gaussian
Kernel scale	6.385
Box constraint	956.32

**Table 3:** Selected classification model 2 training options after fine tuning.

Parameter	
Optimization function	Stochastic gradient descent with momentum (sgdm)
Epochs	12
Mini-batch	30
Learning rate	0.001
Gradient threshold	1.00
Shuffle	Every epoch

**Table 4:** Original and selected datasets’ class distribution.

Class	Original Data		Selected Data	
	Image Stimulus	Emotional Recall	Image Stimulus	Emotional Recall
Anger	24	24	19	19
Disgust	24	24	19	19
Fear	24	24	19	19
Happiness	24	24	19	19
Neutral	24	24	19	19
Sadness	24	24	19	19
Surprise	24	24	19	19
Rest	168	168	133	133
Total	336	336	266	266

**Table 5:** Original dataset arousal level distribution.

Arousal Level	1	2	3	4	5	6	7	8	9
	Low			Mid			High		
Total observations	24	26	34	29	51	41	31	19	11
	84			121			61		
Training set	78			105			57		
Testing set	6			16			4		

**Table 6:** Mean TP classification accuracy over the 5 folds and classes for each model on the given dataset. Values in bold represent the best overall performance.

Model	Accuracy (%)
K-Nearest Neighbors	91.20
Ensemble	91.20
Discriminant	90.90
Shallow Neural Network	92.60
Naïve Byes	83.30
Support Vector Machine	**94.19**

**Table 7:** Ablation results of the mean TP classification accuracy over all 5 folds and classes for each architecture on the given dataset. Values in bold represent the best overall performance.

Architecture	Accuracy (%)
AlexNet	28.61
GoogleNet	39.10
EfficientNetb0	46.99
VGG16	19.88
SqueezeNet	37.61
ResNet50	**51.14**

## Data Availability

The data used in this article are unavailable due to privacy and ethical restrictions. Data sharing is not applicable to this article.

## Appendix A

Algorithm A1 describes the steps performed in this study to remove outliers from the raw ECG signal for each emotional stimulus response.

**Algorithm A1:** Algorithm For Raw ECG Signal Outlier Removal	
	***Function:*** ECG_Outlier_Removal(ECG, Sampling Frequency)
	***Input:*** ECG signal of emotional stimulus response with outliers
	***Output:*** ECG signal with outliers removed
**1**	S_Freq **←** Sampling Frequency, **st** ← 1, **et** ← S_Freq, Factor_1 **←** 2.5
**2**	**for i** in size(ECG)/S_Freq
**3**	Sig_w ← ECG[**st:et**]
**4**	Min_w[**i**] ← **min**(Sig_w), Max_w[**i**] ← **max**(Sig_w)
**5**	**st** ← **st** + S_Freq, **et** ← **et** + S_Freq
**6**	st_Mat[**i**] ← **st**, et_Mat[**i**] ← **et**
**7**	**end for**
**8**	Min_avg ← mean(Min_w), Max_avg ← mean(Max_w)
**9**	Bool_Out_Min ← Min_w < Factor_1 * Min_avg
**10**	Bool_Out_Max ← Max_w > Factor_1 * Max_avg
**11**	Col_min ← find(Bool_Out_Min), Col_max ← find(Bool_Out_Max)
**12**	Sec_Rem_Mat ← vertical_stack(Col_min, Col_max)
**13**	**for j** in size(Sec_Rem_Mat)
**14**	Rs_C ← Sec_Rem_Mat[**j**]
**15**	**if** Rs_C = 1 or Rs_C = 2
**16**	St_c ← st_Mat[Rs_C], Et_c ← et_Mat[Rs_C]
**17**	St_c_p ← st_Mat[Rs_C + 1], Et_c_p ← et_Mat[Rs_C + 1]
**18**	**else**
**19**	St_c ← st_Mat[Rs_C − 1], Et_c ← et_Mat[Rs_C − 1]
**20**	St_c_p ← st_Mat[Rs_C − 2], Et_c_p ← et_Mat[Rs_C − 2]
**21**	**end if**
**22**	ECG[St_c:Et_c] ← ECG[St_c_p:Et_c_p]
**23**	**end for**

## References

[1] Grifantini K. (2020). Detecting Faces, Saving Lives. IEEE Pulse.

[2] Arabian H., Wagner-Hartl V., Geoffrey Chase J., Möller K. Facial Emotion Recognition Focused on Descriptive Region Segmentation. Proceedings of the 2021 43rd Annual International Conference of the IEEE Engineering in Medicine Biology Society (EMBC).

[3] Committee on Children with Disabilities (2001). The Pediatrician’s Role in the Diagnosis and Management of Autistic Spectrum Disorder in Children. Pediatrics.

[4] Tebartz van Elst L., Fangmeier T., Schaller U.M., Hennig O., Kieser M., Koelkebeck K., Kuepper C., Roessner V., Wildgruber D., Dziobek I. (2021). FASTER and SCOTT&EVA Trainings for Adults with High-Functioning Autism Spectrum Disorder (ASD): Study Protocol for a Randomized Controlled Trial. Trials.

[5] Rylaarsdam L., Guemez-Gamboa A. (2019). Genetic Causes and Modifiers of Autism Spectrum Disorder. Front. Cell. Neurosci..

[6] Mehrabian A., Mortensen C.D. (2017). Communication without Words. Communication Theory.

[7] Xie Z., Yadav S., Jo A. (2021). The Association between Electronic Wearable Devices and Self-Efficacy for Managing Health: A Cross Sectional Study Using 2019 HINTS Data. Health Technol..

[8] Wagner B.E., Folk A.L., Hahn S.L., Barr-Anderson D.J., Larson N., Neumark-Sztainer D. (2021). Recreational Screen Time Behaviors during the COVID-19 Pandemic in the U.S.: A Mixed-Methods Study among a Diverse Population-Based Sample of Emerging Adults. Int. J. Environ. Res. Public Health.

[9] Boucsein W. (2012). Electrodermal Activity.

[10] Boucsein W., Backs R.W. (2000). Engineering Psychophysiology as a Discipline: Historical and Theoretical Aspects. Engineering Psychophysiology: Issues and Applications.

[11] Birkle J., Weber R., Möller K., Wagner-Hartl V. Psychophysiological Parameters for Emotion Recognition–Conception and First Evaluation of a Measurement Environment. Proceedings of the 5th International Conference on Intelligent Human Systems Integration; Integrating People and Intelligent Systems.

[12] Dawson M.E., Schell A.M., Filion D.L. (2007). The Electrodermal System. Handbook of Psychophysiology.

[13] Fozzard H.A. (1991). The ECG and the Single Channel. J. Electrocardiol..

[14] Ashley E.A., Niebauer J. (2004). Conquering the ECG. Cardiology Explained.

[15] Schmid R., Saat S.M., Möller K., Wagner-Hartl V. Induction Method Influence on Emotion Recognition Based on Psychophysiological Parameters. Proceedings of the Intelligent Human Systems Integration (IHSI 2023): Integrating People and Intelligent Systems.

[16] Bradley M.M., Lang P.J. (1994). Measuring Emotion: The Self-Assessment Manikin and the Semantic Differential. J. Behav. Ther. Exp. Psychiatry.

[17] PubMed. https://pubmed.ncbi.nlm.nih.gov/.

[18] Kakuba S., Poulose A., Han D.S. (2022). Attention-Based Multi-Learning Approach for Speech Emotion Recognition with Dilated Convolution. IEEE Access.

[19] Yan Y., Shen X. (2022). Research on Speech Emotion Recognition Based on AA-CBGRU Network. Electronics.

[20] Khaireddin Y., Chen Z. (2021). Facial Emotion Recognition: State of the Art Performance on FER2013. arXiv.

[21] Challenges in Representation Learning: Facial Expression Recognition Challenge. https://kaggle.com/c/challenges-in-representation-learning-facial-expression-recognition-challenge.

[22] Mehendale N. (2020). Facial Emotion Recognition Using Convolutional Neural Networks (FERC). SN Appl. Sci..

[23] Zhao X., Liang X., Liu L., Li T., Han Y., Vasconcelos N., Yan S. (2017). Peak-Piloted Deep Network for Facial Expression Recognition. arXiv.

[24] Szegedy C., Liu W., Jia Y., Sermanet P., Reed S., Anguelov D., Erhan D., Vanhoucke V., Rabinovich A. (2014). Going Deeper with Convolutions. arXiv.

[25] Kim J.H., Poulose A., Han D.S. (2021). The Extensive Usage of the Facial Image Threshing Machine for Facial Emotion Recognition Performance. Sensors.

[26] Canal F.Z., Müller T.R., Matias J.C., Scotton G.G., de Sa Junior A.R., Pozzebon E., Sobieranski A.C. (2022). A Survey on Facial Emotion Recognition Techniques: A State-of-the-Art Literature Review. Inf. Sci..

[27] Karnati M., Seal A., Bhattacharjee D., Yazidi A., Krejcar O. (2023). Understanding Deep Learning Techniques for Recognition of Human Emotions Using Facial Expressions: A Comprehensive Survey. IEEE Trans. Instrum. Meas..

[28] Shukla J., Barreda-Ángeles M., Oliver J., Nandi G.C., Puig D. (2021). Feature Extraction and Selection for Emotion Recognition from Electrodermal Activity. IEEE Trans. Affect. Comput..

[29] Al Machot F., Elmachot A., Ali M., Al Machot E., Kyamakya K. (2019). A Deep-Learning Model for Subject-Independent Human Emotion Recognition Using Electrodermal Activity Sensors. Sensors.

[30] Veeranki Y.R., Ganapathy N., Swaminathan R. (2021). Electrodermal Activity Based Emotion Recognition Using Time-Frequency Methods and Machine Learning Algorithms. Curr. Dir. Biomed. Eng..

[31] Shu L., Yu Y., Chen W., Hua H., Li Q., Jin J., Xu X. (2020). Wearable Emotion Recognition Using Heart Rate Data from a Smart Bracelet. Sensors.

[32] Greco A., Marzi C., Lanata A., Scilingo E.P., Vanello N. Combining Electrodermal Activity and Speech Analysis towards a More Accurate Emotion Recognition System. Proceedings of the 2019 41st Annual International Conference of the IEEE Engineering in Medicine and Biology Society (EMBC).

[33] Du G., Long S., Yuan H. (2020). Non-Contact Emotion Recognition Combining Heart Rate and Facial Expression for Interactive Gaming Environments. IEEE Access.

[34] Fernández-Aguilar L., Martínez-Rodrigo A., Moncho-Bogani J., Fernández-Caballero A., Latorre J.M., Ferrández Vicente J.M., Álvarez-Sánchez J.R., de la Paz López F., Toledo Moreo J., Adeli H. (2019). Emotion Detection in Aging Adults Through Continuous Monitoring of Electro-Dermal Activity and Heart-Rate Variability. Understanding the Brain Function and Emotions.

[35] Zhang Z., Song Y., Cui L., Liu X., Zhu T. (2016). Emotion Recognition Based on Customized Smart Bracelet with Built-in Accelerometer. PeerJ.

[36] Soleymani M., Lichtenauer J., Pun T., Pantic M. (2012). A Multimodal Database for Affect Recognition and Implicit Tagging. IEEE Trans. Affect. Comput..

[37] Koelstra S., Muhl C., Soleymani M., Lee J.-S., Yazdani A., Ebrahimi T., Pun T., Nijholt A., Patras I. (2012). DEAP: A Database for Emotion Analysis Using Physiological Signals. IEEE Trans. Affect. Comput..

[38] Lin W., Li C. (2023). Review of Studies on Emotion Recognition and Judgment Based on Physiological Signals. Appl. Sci..

[39] Chalmers T., Hickey B.A., Newton P., Lin C.-T., Sibbritt D., McLachlan C.S., Clifton-Bligh R., Morley J., Lal S. (2021). Stress Watch: The Use of Heart Rate and Heart Rate Variability to Detect Stress: A Pilot Study Using Smart Watch Wearables. Sensors.

[40] ECG and Activity Sensor—EcgMove 4—Movisens GmbH. https://www.movisens.com/en/products/ecg-sensor/.

[41] EDA and Activity Sensor—EdaMove 4. https://www.movisens.com/en/products/eda-and-activity-sensor/.

[42] Shaffer F., Ginsberg J.P. (2017). An Overview of Heart Rate Variability Metrics and Norms. Front. Public Health.

[43] Rosner B. (1983). Percentage Points for a Generalized ESD Many-Outlier Procedure. Technometrics.

[44] Akima H. (1970). A New Method of Interpolation and Smooth Curve Fitting Based on Local Procedures. J. ACM.

[45] Detect and Replace Outliers in Data—MATLAB Filloutliers. https://www.mathworks.com/help/matlab/ref/filloutliers.html.

[46] Baek H.J., Cho C.-H., Cho J., Woo J.-M. (2015). Reliability of Ultra-Short-Term Analysis as a Surrogate of Standard 5-Min Analysis of Heart Rate Variability. Telemed. J. E Health.

[47] Lomb N.R. (1976). Least-Squares Frequency Analysis of Unequally Spaced Data. Astrophys. Space Sci..

[48] Morlet J., Chen C.H. (1983). Sampling Theory and Wave Propagation. Issues in Acoustic Signal—Image Processing and Recognition.

[49] Cervantes J., Garcia-Lamont F., Rodríguez-Mazahua L., Lopez A. (2020). A Comprehensive Survey on Support Vector Machine Classification: Applications, Challenges and Trends. Neurocomputing.

[50] Awad M., Khanna R., Awad M., Khanna R. (2015). Support Vector Machines for Classification. Efficient Learning Machines: Theories, Concepts, and Applications for Engineers and System Designers.

[51] He K., Zhang X., Ren S., Sun J. (2015). Deep Residual Learning for Image Recognition. arXiv.

[52] Krizhevsky A., Sutskever I., Hinton G.E. (2017). ImageNet Classification with Deep Convolutional Neural Networks. Commun. ACM.

[53] Simonyan K., Zisserman A. (2015). Very Deep Convolutional Networks for Large-Scale Image Recognition. arXiv.

[54] Tan M., Le Q.V. (2020). EfficientNet: Rethinking Model Scaling for Convolutional Neural Networks. arXiv.

[55] Iandola F.N., Han S., Moskewicz M.W., Ashraf K., Dally W.J., Keutzer K. (2016). SqueezeNet: AlexNet-Level Accuracy with 50x Fewer Parameters and <0.5MB Model Size 2016. arXiv.

